# Studying medicine from home: an cross-sectional study on the impact of online education in Romanian medical students

**DOI:** 10.1192/j.eurpsy.2023.1254

**Published:** 2023-07-19

**Authors:** S. Zaharia, S.-A. Simtea, T.-C. Ionescu, C. Tudose

**Affiliations:** 1 “Prof. Dr. Alexandru Obregia” Clinical Psychiatric Hospital; 2 “Carol Davila” University of Medicine and Pharmacy, Bucharest, Romania

## Abstract

**Introduction:**

Despite the literature regarding the impact of the COVID-19 pandemic on education, there is little research that specifically targets medical students and their relationship with online courses in regards to engagement and feelings of inadequacy.

**Objectives:**

This cross-sectional study aims to explore such questions by evaluating a small (N=169) sample of Romanian medical students and applying self-reporting questionnaires in order to quantify subjective levels of burnout and imposter phenomenon

**Methods:**

Responders filled an online survey with question regarding miscellaneous socio-demographic factors, alont with the Academic Burnout Scale (ABS), Clarence Imposter Phenomenon Scale (CIPS) and Ohio Resilience Scale (ORS). Results were collected and analysed for subsequent correlations.

**Results:**

Predictably, respondents already in favour with online courses showed less signs of burnout and higher levels of resilience. While higher-year students preferred online courses, particularly final year students, it was lower-year students who showed higher level of resilience and lower burnout and imposter phenomenon levels, possibly suggesting a more profound impact of online education on students in clinical rotations, as opposed to pre-clinical ones. No statistically significant correlations were found between socio-demographic factors and the self-reported ratings, showing that feelings of burnout and imposter phenomenon were equally distributed among genders.

**Conclusions:**

The results of this study present a snapshot into the opinions of future Romanian medical professionals on their own education and, in spite of its methodological limitations, can function as a starting point for deeper and more exhaustive inquiry regarding medical education during COVID-19 times.

**Disclosure of Interest:**

None Declared

